# Retraction of article: Effectiveness and safety of Bifidobacterium in preventing dental caries: a systematic review and meta-analysis

**DOI:** 10.2340/aos.v84.43064

**Published:** 2025-08-19

**Authors:** Siyuan Hao, Jiahe Wang, Yan Wang

**Affiliations:** a State Key Laboratory of Oral Diseases & National Clinical Research Center for Oral Diseases, West China Hospital of Stomatology, Sichuan University, Chengdu, P. R. China

Retraction notice for article: https://doi.org/10.1080/00016357.2021.1921259

Due to an unintentional publication of a similar article in Chinese language from the same group, the authors have requested retraction of the article: Siyuan Hao, Jiahe Wang, Yan Wang. Effectiveness and safety of Bifidobacterium in preventing dental caries: a systematic review and meta-analysis. Acta Odontol Scand.

The reason for the retraction is that the authors discovered the data in this article is almost identical to that in a previously published Chinese article by their team (doi:10.12016/j.issn.2096-1456.2021.04.004). This issue arose partially due to poor communication among the authors. Yan Wang mistakenly assumed that the studies included in the final analysis were significantly different because different databases were searched for the Chinese and English articles. All the authors deeply regret these errors and unanimously agree to this retraction. They sincerely apologize to everyone involved with this manuscript, including the editors, reviewers, and other staff members.

